# Quick, Selective and Reversible Photocrosslinking Reaction between 5-Methylcytosine and 3-Cyanovinylcarbazole in DNA Double Strand

**DOI:** 10.3390/ijms14035765

**Published:** 2013-03-12

**Authors:** Kenzo Fujimo, Kaoru Konishi-Hiratsuka, Takashi Sakamoto

**Affiliations:** 1 School of Materials Science, Japan Advanced Institute of Science and Technology, 1-1 Asahi-dai, Nomi, Ishikawa 923-1292, Japan; E-Mails: kaoru-h@jaist.ac.jp (K.K.-H.); tsakamo@jaist.ac.jp (T.S.); 2 Research Center for Bio-Architecture, Japan Advanced Institute of Science and Technology, 1-1 Asahi-dai, Nomi, Ishikawa 923-1292, Japan

**Keywords:** photocrosslinking, 5-methylcytosine, 3-cyanovinylcarbazole nucleoside

## Abstract

Selective photocrosslinking reaction between 3-cyanovinylcarbazole nucleoside (^CNV^K) and 5-methylcytosine (^m^C), which is known as epigenetic modification in genomic DNA, was developed. The reaction was completely finished within 5 s of 366 nm irradiation, and the rate of this photocrosslinking reaction was *ca.* 30-fold higher than that in the case of unmodified normal cytosine. There were no significant differences in the thermodynamic parameters and the kinetics of hybrid formation of oligonucleotide (ODN) containing ^CNV^K and its complementary ODN containing C or ^m^C at the photocrosslinking site, and suggesting that the quick and selective photoreaction has potential for the selective detection of ^m^C in the DNA strand via the photocrosslinking reaction.

## 1. Introduction

5-Methylcytosine (^m^C) is the most abundant epigenetic modification in genomic DNA and plays a role in the epigenetic regulation of gene expression, genomic imprinting, cell differentiation, and tumorigenesis [1,2]. Therefore, the development of methodology for the detection of ^m^C in genomic DNA is required. Various methods have been developed for detecting ^m^C based on chemical and enzymatic concepts [3–5]. We previously reported on photoresponsive synthetic oligonucleotides (ODN(s)) containing 5-vinyl-2′-deoxyuridine (^V^U) derivatives [6–10] that can photoligate to target DNA via [2 + 2] photocycloaddition with a pyrimidine base in target DNA. In particular, ^V^U derivatives having a hydrophobic group, such as cyano [11], cyclohexyl [12], cyclopentyl [12], and aromatic group [13] were selectively photoligated to ODNs containing ^m^C because of the hydrophobic interaction between the 5-methyl group on target ^m^C and hydrophobic moiety tethered to ^V^U. However, the photoreactivity of these ^V^U derivatives was low, and the photoreaction requires long photoirradiation time, approximately 10 min.

On the other hand, we previously reported that ODNs having 3-cyanovinylcarbazole nucleoside (^CNV^K) can photocrosslink to complementary DNA strand via [2 + 2] photocycloaddition between ^CNV^K and pyrimidine base in complementary strand with 1 s of 366 nm irradiation [14–18]. If the rapid photocrosslinking reaction of ^CNV^K had the selectivity for ^m^C like ^V^U derivatives having hydrophobic group, rapid and selective detection of ^m^C in the target DNA sequence will be possible. In addition, such a quick and selective photocrosslinking reaction might be a basic reaction for *in situ*^m^C mapping and imaging in genomic DNA.

In this manuscript, we describe the ^m^C selective photocrosslinking reaction of ODNs containing ^CNV^K and also describe what causes the selective photocrosslinking reaction from the viewpoint of the difference of thermodynamic, kinetic and electronic properties of the ODN duplex containing ^CNV^K and ^m^C or C.

## 2. Results and Discussion

### 2.1. Reactivity and Selectivity of the Photocrosslinking Reaction to ^m^C

**Figure 1:**
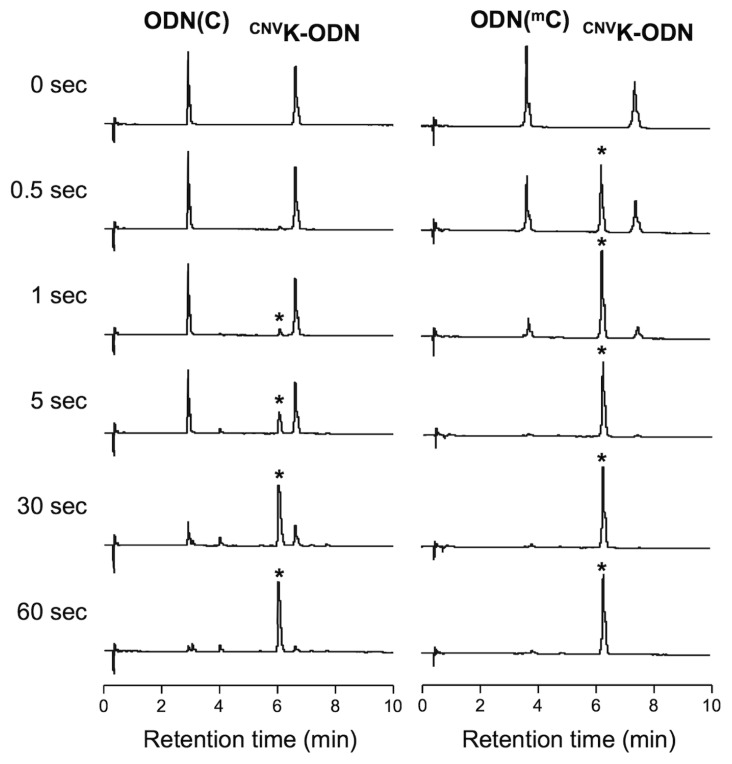
UPLC analysis of the photocrosslinking reaction between ^CNV^K-ODN and ODN (C) (**left**) or ODN (^m^C) (**right**). [^CNV^K-ODN] = [ODN(C) or (^m^C)] = 5 μM in 50 mM Na-Cacodylate buffer (pH 7.4) containing 100 mM NaCl. Photoirradiation (366 nm) was performed at 20 °C. Asterisks indicate peaks identical to the photocrosslinked duplexes.

**Figure 2:**
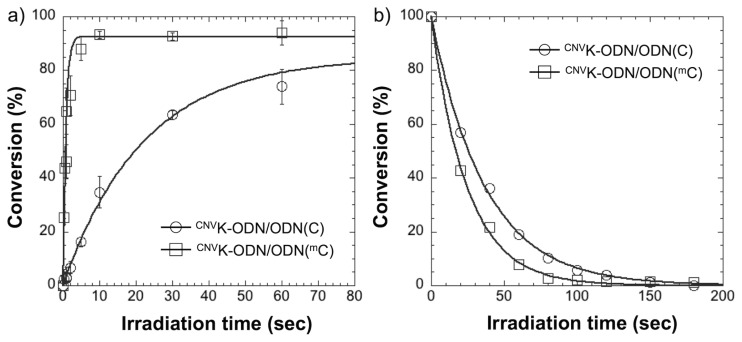
(**a**) Time course of the photocrosslinking reaction between ^CNV^K-ODN and ODN(C) or ODN (^m^C) with 366 nm irradiation. [^CNV^K-ODN] = [ODN(C) or (^m^C)] = 5 μM in 50 mM Na-Cacodylate buffer (pH 7.4) containing 100 mM NaCl. Photoirradiation (366 nm) was performed at 20 °C; (**b**) Time course of the photosplitting reaction of the crosslinked duplexes consist of ^CNV^K-ODN and ODN(C) or ODN(^m^C) with 312 nm irradiation. [Duplex] = 5 μM in 50 mM Na-Cacodylate buffer (pH 7.4) containing 100 mM NaCl. Photoirradiation (312 nm) was performed at 60 °C.

To assess the photoreactivity of ^CNV^K to ^m^C, at first, we performed the photocrosslinking reaction between ^CNV^K-ODN (5′-TGCG^CNV^KTCGT-3′) and ODN(^m^C) (5′-ACGAG^m^CGCA-3′) by a 366 nm irradiation and then analyzed by ultra-high performance liquid chromatography (UPLC). As shown in Figure 1, right, the peaks of ^CNV^K-ODN and ODN(^m^C) were decreased and completely disappeared within 5 s irradiation, and a new peak, which was identical to the photodimer consists of ^CNV^K-ODN and ODN(^m^C) ([(M + H)^+^], Calcd. 5558.06, found 5557.60), clearly appeared. By the MALDI-TOF-MS analysis of the product after the nuclease and phosphatase treatment of the photocrosslinked duplex, the photoadduct consists of ^CNV^K and 5-methyldeoxycytidine ([(M + Na)^+^], Calcd. 598.23, found 598.89) was detected, suggesting that the reaction occurred via the [2 + 2] photocycloaddition between ^CNV^K and ^m^C, the same as in the case of T [14]. In the case of ODN(C) (5′-ACGAGCGCA-3′) (Figure 1, left), the photocrosslinking reaction between ^CNV^K-ODN and ODN(C) also occurred, although the reaction rate was low compared with the case of ODN(^m^C). To quantitatively evaluate the photoreactivity of ^CNV^K-ODN to ODN(^m^C) or ODN(C), the time course of the conversion of these photocrosslinking reactions was monitored by UPLC (Figure 2a) and the time to reach 50% conversion (T_50_) is listed in Table 1. In the case of ^CNV^K-ODN/ODN(^m^C) duplex, the T_50_ value was *ca*. 30-fold lower than that in the case of ^CNV^K-ODN/ODN(C) duplex, suggesting that the C5 methylation of C accelerate the photocrosslinking reaction. To confirm the generality of the acceleration effect on the photocrosslinking reaction of ^CNV^K to pyrimidine base, photocrosslinking reactions using ^CNV^K-ODN(A) (5′-TGCA^CNV^KTCGT-3′) and ODN(T) (5′-ACGAGTGCA-3′) or ODN(U) (5′-ACGAGUGCA-3′) were performed. As shown in Table 1 and Figure S1, no significant difference dependent on C5 methylation was observed, suggesting that the presence of a methyl group on the C5 position of pyrimidine base was not a general reason for the acceleration effect. As shown in Table 1, Figures 2 and S2, the photosplitting reaction of photodimer consisting of ^CNV^K-ODN/ODN(^m^C) was induced by 80 s of 312 nm irradiation, indicating that the photocrosslinked dimer can be easily reversed into the original two ODNs. The quick, reversible and selective photocrosslinking reaction for ^m^C has the potential to be a key reaction for the selective detection of methylated site on genomic DNA. Indeed, we demonstrated that our photocrosslinking strategy could detect about 10% methylation of C in target ODN with 1 s of photoirradiation (Figure S3). Further optimization of the detection condition or photoirradiation time would improve the detection limit of the method. Furthermore, as the deamination reaction of the C4 amino group of C and ^m^C is dramatically accelerated when these bases make cyclobutane type photodimer [19–21], and as we previously found that the reversible photocrosslinking of ^CNV^K to C accelerate the C to U transition in DNA and RNA strand [15,16], the reversible photocrosslinking reaction would be a key reaction for site specific induction of ^m^C to T mutation in DNA strand.

### 2.2. Thermodynamics and Kinetics of the Hybridization between ^CNV^K-ODN and ODN(^m^C)

**Figure 3:**
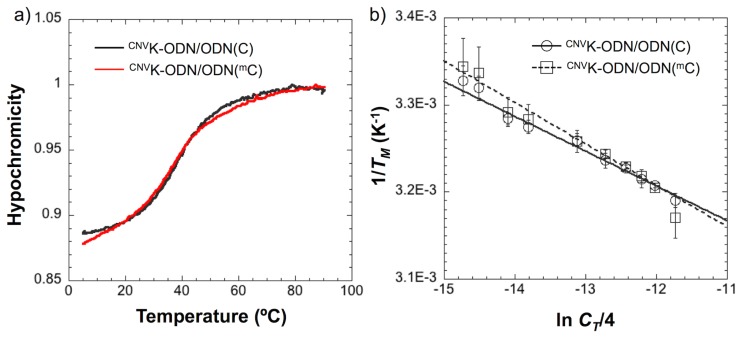
(**a**) UV melting curves of the duplexes consist of ^CNV^K-ODN and ODN(C) or ODN(^m^C). [duplex] = 10 μM in 50 mM Na-cacodylate buffer (pH 7.4) containing 100 mM NaCl. Absorbance was monitored at 260 nm; (**b**) 1/*T*_M_*vs.* ln(*C*_T_/4) plots of the hybridization between ^CNV^K-ODN and ODN(C) or ODN(^m^C).

**Figure 4:**
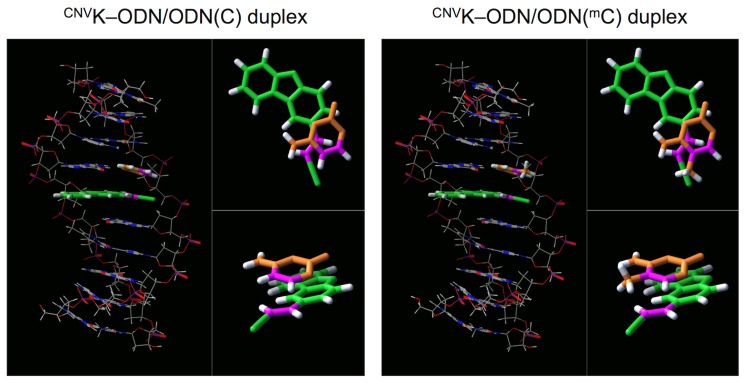
(**a**) Predicted structure of ^CNV^K-ODN/ODN(C) duplex; (**b**) Predicted structure of ^CNV^K-ODN/ODN(^m^C) duplex. These structures were calculated by MacroModel ver. 8.1 with Amber* force field.

To reveal the reason why the ^CNV^K-ODN has selectivity for ODN(^m^C), at first, the melting profiles of the duplexes consisting of ^CNV^K-ODN and ODN(^m^C) or ODN(C) were measured. As shown in Figure 3a and Table 2, no significant difference in the melting temperature (*T*_M_) of each duplexes was observed, although in the case of ^CNV^K-ODN/ODN(C), sharper transition compared to ^CNV^K-ODN/ODN(^m^C) was observed. This suggests that there are entropic differences between the hybridization of each duplex. To evaluate the thermodynamic parameters of these duplexes, van’t-Hoff experiments were performed according to a method in the literature [22] (Figure 3b, Table 2). The Δ*G°*_37_ of these duplexes were similar, indicating that the stability of these duplexes is not different from each other. In general, the C5 methylation of cytosine causes stabilization of DNA duplex (ΔΔ*G°*_37_ = 0.5 kcal/mol) because of the increase of the hydrophobic stacking interaction with neighboring bases [23], however, in our case, such a stabilization effect was not observed. As the 3-cyanovinylcarbazole moiety in the ^CNV^K-ODN/ODN(^m^C) duplex disturbs the stacking interaction around the ^m^C (Figure 4), it seems that the stabilization effect caused by the hydrophobicity of the ^m^C was cancelled and was not observed in our case. On the other hand, Δ*H°* and Δ*S°* of ^CNV^K-ODN/ODN(C) were smaller than that in the case of ^CNV^K-ODN/ODN(^m^C) (19% and 23%, respectively), however, the selective photoreaction of ^CNV^K toward ^m^C could not be explained clearly from the thermodynamic study. Kinetic experiments for the hybridization of ^CNV^K-ODN/ODN(C) and ^CNV^K-ODN/ODN(^m^C) were also conducted by surface plasmon resonance experiments. Unfortunately, no significant difference between ^CNV^K-ODN/ODN(C) and ^CNV^K-ODN/ODN(^m^C) was observed (Table 3). We concluded that the thermodynamic and also kinetic properties of the duplex formation were not major reasons for the selective photocrosslinking reaction for ^m^C.

### 2.3. Extinction Coefficient of ^CNV^K in DNA Duplex

**Figure 5:**
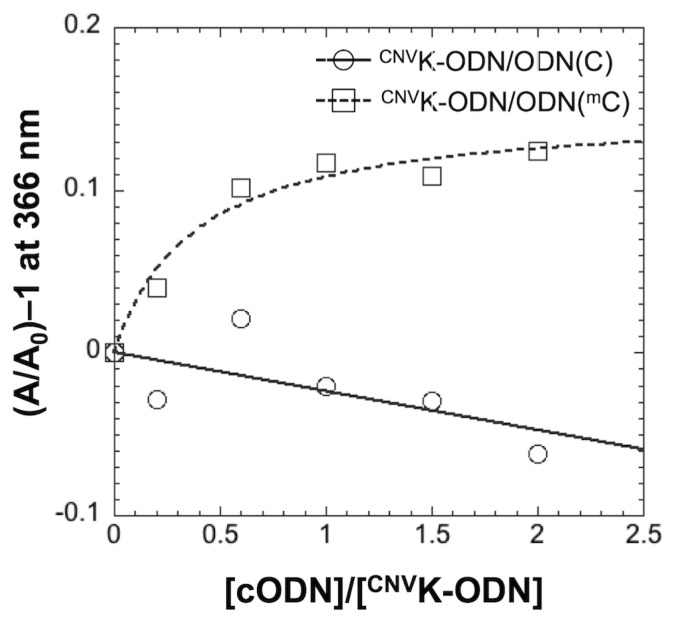
UV titration analysis of ODN(C) and ODN(^m^C) to ^CNV^K-ODN. Absorbance was monitored at 366 nm. [^CNV^K-ODN] = 6 μM in 50 mM Na-Cacodylate buffer (pH 7.4) containing 100 mM NaCl. Experiments were performed at 20 °C.

To clear the reasons for the selective photocrosslinking reaction for ^m^C, finally, we performed a UV titration experiment of the ODN(C) or ODN(^m^C) to the ^CNV^K-ODN. Absorbance of 3-cyanovinylcarbazole moiety (366 nm) was monitored at various concentrations of ODN(C) or ODN(^m^C). As shown in Figure 5, in the case of ODN(C), absorbance at 366 nm was decreased with the increase of the complementary strand, however, absorbance at 366 nm was increased and the change was saturated at the equimolar concentration in the case of ODN(^m^C), suggesting that the electronic state of the 3-cyanovinylcarbazole moiety was different between ^CNV^K-ODN/ODN(C) and ^CNV^K-ODN/ODN(^m^C) duplexes. The difference in the extinction coefficient of ^CNV^K in each duplex means that the excitation yield of the ^CNV^K was different between the case of each duplex. Since the ^CNV^K-ODN/ODN(^m^C) duplex has the higher extinction coefficient compared to ^CNV^K-ODN/ODN(C) duplex, the selectivity of the photocrosslinking reaction for ^m^C can clearly be explained by this phenomenon. Moreover, in the case of ^CNV^K-ODN(A), the *T*_50_ of the photocrosslinking reaction with ODN(T) was *ca*. 40-fold lower than that of ^CNV^K-ODN/ODN(C) (Table 1) and the absorbance at 366 nm was increased 20% by the addition of ODN(T), suggesting that the high reactivity observed in the photocrosslinking with T and ^m^C can partly explained by the excitation yield of the ^CNV^K in DNA double strand.

## 3. Experimental Section

### 3.1. Preparation of ODNs

ODN sequences were synthesized by the conventional phosphoramidite method by using an Applied Biosystems 3400 DNA synthesizer. The coupling efficiency was monitored with a trityl monitor. The coupling efficiency of crude mixture of ^CNV^K was 97% yield. The coupling time for ^CNV^K was 999 s. They were deprotected by incubation with 28% ammonia for 4 h at 65 °C and were purified on a InertSustain™ C18 column (GL Science, 5 μm, 10 × 150 mm) by reverse phase HPLC; elution was with 0.05 M ammonium formate containing 3%–20% CH_3_CN, linear gradient (30 min) at a flow rate of 3.0 mL/min. Preparation of oligonucleotides was confirmed by MALDI-TOF-MS analysis: ^CNV^K-ODN [(M + H)^+^], calcd. 2812.53, found 2812.20, ^CNV^K-ODN(A) [(M + H)^+^], calcd. 2796.53, found 2795.41, ODN(C) [(M + H)^+^], calcd. 2732.52, found 2732.93, ODN(^m^C) [(M + H)^+^], calcd. 2746.54, found 2746.93, ODN(U) [(M + H)^+^], calcd. 2733.51, found 2733.96, ODN(T) [(M + H)^+^], calcd. 2747.52, found 2747.94.

### 3.2. Photoirradiation and UPLC Analysis

To avoid the absorption saturation, *i.e.*, inner filter effect, the optical density of the sample at 366 nm was kept below 0.1. Photoirradiation was performed with an LED lamp (ZUV, 366 nm, 1600 mW/cm^2^, Omron Corporation, Kyoto, Japan) and a transilluminator (312 nm, Funakoshi) on an aluminum block incubator or water bath. The photoirradiated samples were analyzed with a UPLC system (Aquity, Waters, Milford, MA, USA) equipped with BEH Shield RP18 column (1.7 μm, 2.1 × 50 mm, elution was with 0.05 M ammonium formate containing 1%–10% CH_3_CN, linear gradient (10 min) at a flow rate of 0.4 mL/min, 60 °C).

### 3.3. Enzymatic Digestion, HPLC and MALDI-TOF-MS Analysis

The enzymatic digestion was carried out with the treatment of snake venom phosphodiesterase (0.2 U), P1 nuclease (1 U) and calf intestine alkaline phosphatase (20 U) in 50 mM Tris-HCl buffer (pH 9.0) containing 1 mM MgCl^2^, 0.1 mM ZnCl^2^ and 1 mM Spermidin at 37 °C for 5 h. After the purification of the photoadducts by reversed phase HPLC with InertSustain™ C18 (GL Science, 5 μm, 10 × 150 mm, elution was with 0.05 M ammonium formate containing 1%–50% CH_3_CN, linear gradient (50 min) at a flow rate of 3 mL/min), the molecular masses were analyzed with a MALDI-TOF-Mass spectrometer (Voyager DE-Pro-SF, Applied Biosystems, Foster City, CA, USA).

### 3.4. Thermodynamic and Kinetic Analysis of the Hybridization

Thermodynamic parameters were obtained by the following equations according to a method in the literature:

(1) $$T_{\text{M}}^{-1}=\left[R\;\text{ln }(\frac{C_{\text{T}}}{4})+\Delta S^{0}\right]/\Delta H^{\text{O}}$$

where *T*_M_ is the melting temperature of duplex, *ΔS°* is entropy and *ΔH°* is the entharpy of duplex formation, respectively. *R* is the gas constant and *C*_T_ is the total strand concentration. *T*_M_ was measured at various concentrations of duplex (0.5–16 μM in 50 mM Na-cacodylate buffer (pH 7.4) containing 100 mM NaCl) by a spectrophotometer (V-630bio, Jasco, Tokyo, Japan) equipped with a temperature controller.

Kinetic parameters of the hybridization were collected by a surface plasmon resonance biosensor (BIAcore J, GE Healthcare, Buckinghamshire, UK) with avidine chips that having biotine-modified ^CNV^K-ODN on the surface. After the injection of various concentrations of ODN(C) or ODN(^m^C), the sensorgrams were collected. (20 °C, 10 mM HEPES buffer (pH 7.4), 0.15 M NaCl, 3 mM EDTA, 0.005% Tween-20).

## 4. Conclusions

In this study, we found that ODN having ^CNV^K can rapidly photocrosslink to ^m^C in a complementary DNA strand and that the photocrosslinking reaction has a C5 methylation selective manner. As the photocrosslinking reaction was finished within 5 s of 366 nm irradiation, the reaction has the potential to become a key reaction for the selective detection of ^m^C in the DNA strand and for the site specific induction of ^m^C to T mutation. Thermodynamic and kinetic study revealed that the selectivity for ^m^C of this photocrosslinking reaction is not caused by thermodynamics or kinetics of the hybridization. It seems that the conformational difference around the ^m^C and C affects the selectivity of the quick, reversible photocrosslinking reaction. Based on the selective photoreaction toward ^m^C, selective detection on genomic DNA would be possible with *in situ* hybridization technique using fluorescence- or biotin- labeled ODN having ^CNV^K.

## Supplementary Information



## Figures and Tables

**Table 1:** Time to reach 50% conversion (*T*_50_) in the photocrosslinking and the photosplitting reactions of ^CNV^K-ODN.

Duplex	*T*_50_ (s)	

	Photocrosslinking	Photosplitting
^CNV^K-ODN/ODN(C)	19.6	25.5
^CNV^K-ODN/ODN(^m^C)	0.6	17.0
^CNV^K-ODN(A)/ODN(U)	0.6	-
^CNV^K-ODN(A)/ODN(T)	0.5	-

**Table 2:** Thermodynamic parameters of the duplexes consist of ^CNV^K-ODN and ODN(C) or ODN(^m^C).

Duplex	Δ*G°*_37_ kcal/mol	Δ*H°* kcal/mol	Δ*S°* cal/mol K	*T*_M_ °C
^CNV^K-ODN/ODN(C)	−7.7	−49.8	−135	37.9 ± 0.9
^CNV^K-ODN/ODN(^m^C)	−7.6	−41.8	−110	37.4 ± 0.7

**Table 3:** Kinetic parameters of the duplex formation between ^CNV^K-ODN and ODN(C) or ODN(^m^C).

Duplex	*K*_D_ (μM)	*k*_a_ (×10^3^ s^−1^ M^−1^)	*k*_d_ (s^−1^)
^CNV^K-ODN/ODN(C)	2.4 ± 0.14	48	0.11
^CNV^K-ODN/ODN(^m^C)	2.2 ± 0.05	52	0.11
