# Supplementary material for: Quick, Selective and Reversible Photocrosslinking Reaction between 5-Methylcytosine and 3-Cyanovinylcarbazole in DNA Double Strand
Source: Int J Mol Sci. 2013 Mar 12;14(3):5765–74. doi: 10.3390/ijms14035765 (PMC3634424; doi:10.3390/ijms14035765)
Supplement: Supplementary file 1 [file ijms-14-05765-s001.pdf]

Supplementary Information

Figure S1. Time course of the photocrosslinking reaction between ^{CNV}K-ODN(A) and ODN(U) or ODN(T) with 366 nm irradiation. [^{CNV}K-ODN(A)] = [ODN(U) or (T)] = 5 μM in 50 mM Na-Cacodylate buffer (pH 7.4) containing 100 mM NaCl. Photoirradiation (366 nm) was performed at 20 °C.

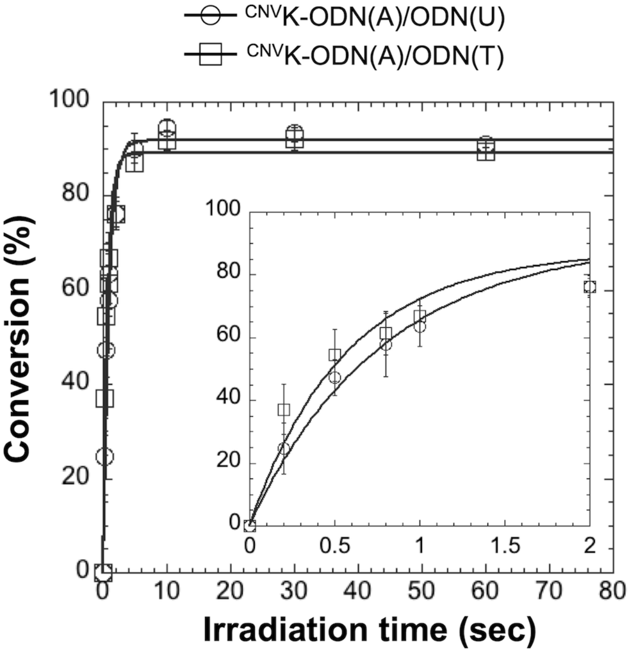

Figure S2. UPLC analysis of the photosplitting reaction of photocrosslinked CNVK-ODN/ODN (C) (left) and CNVK-ODN/ODN (mC) (right) with 312 nm irradiation. [CNVK-ODN] = [ODN(C) or (mC)] = 5 μM in 50 mM Na-Cacodylate buffer (pH 7.4) containing 100 mM NaCl. Photoirradiation (312 nm) was performed at 60 °C.

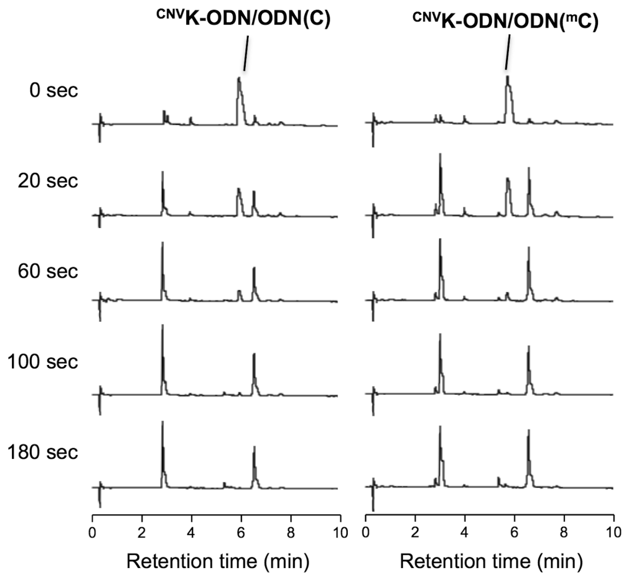

Figure S3. Relative amount of photodimer after the photocrosslinking with the various contents of mC in target ODN. [CNVK-ODN] = 2.5 µM, [ODN(C) + ODN(mC)] = 5 µM in 50 mM Na-Cacodylate buffer (pH 7.4) containing 100 mM NaCl. Photoirradiation (366 nm) was performed for 1 s at 20 °C.

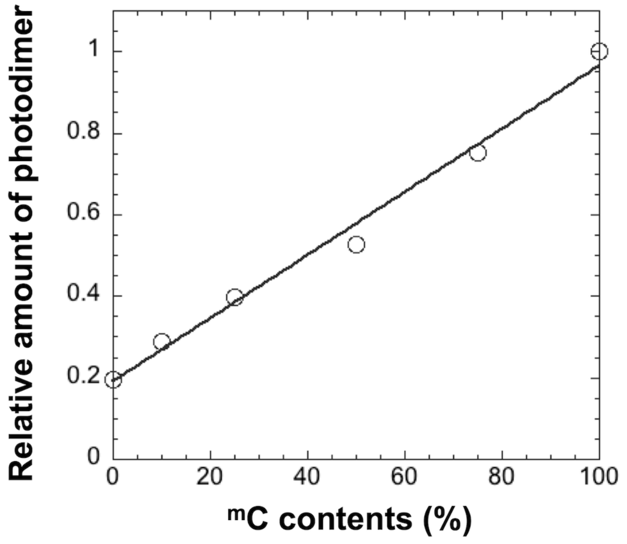

Figure S4. SPR sensorgram of the hybridization between CNVK-ODN and ODN (C) (left) or ODN (mC) (right). [ODN(C) or ODN(mC)] = 0.5, 0.75, 1.0, 2.5, 4, 6, 8, and 10 µM. Measurement were carried out at 20 °C.

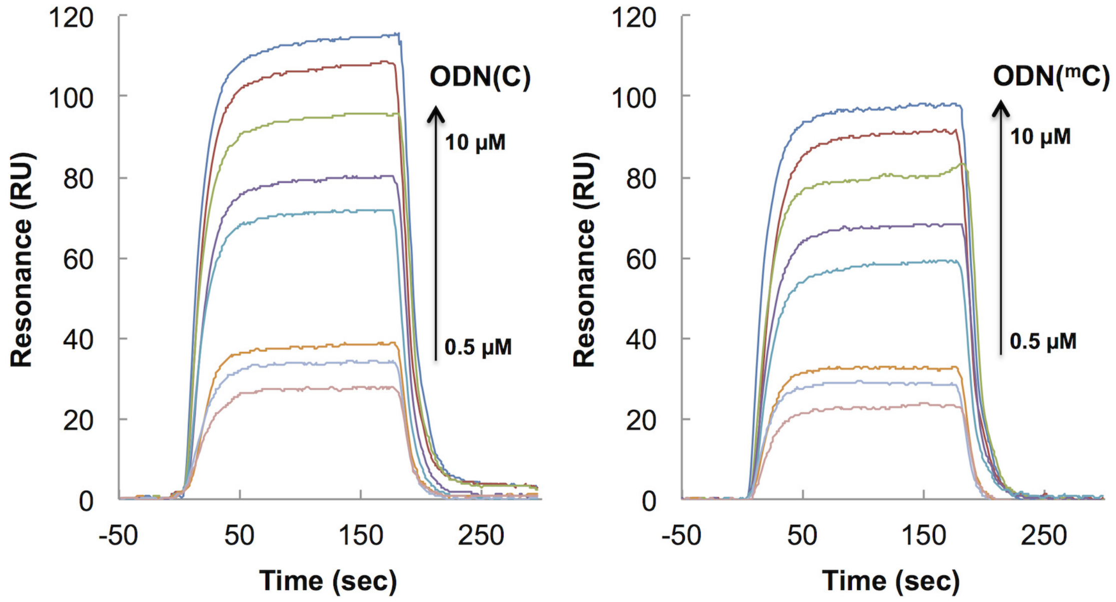

© 2013 by the authors; licensee MDPI, Basel, Switzerland. This article is an open access article distributed under the terms and conditions of the Creative Commons Attribution license (http://creativecommons.org/licenses/by/3.0/).
